# Acrolein target-linked toxicological perturbation and tumor-immune microenvironment heterogeneity in colorectal cancer

**DOI:** 10.3389/fcvm.2026.1929802

**Published:** 2026-10-14

**Authors:** Gongming Cheng, Liuqun Shan, Wenjie Shi, Xucheng Liu, Xuewen Tao, Jianbo Han

**Affiliations:** Department of Gastrointestinal Surgery, The Second Hospital of Nanjing, Affiliated to Nanjing University of Chinese Medicine, Nanjing, Jiangsu, China

**Keywords:** acrolein, colorectal cancer, comparative toxicogenomics database, single-cell RNA-seq, stromal heterogeneity, TCGA, toxicogenomics, tumor immune microenvironment

## Abstract

**Background:**

Acrolein is a highly reactive aldehyde generated endogenously during lipid peroxidation and encountered through environmental and dietary exposures. Although acrolein has established electrophilic, genotoxic, and oxidative-stress effects, the extent to which acrolein-linked gene perturbation is represented in colorectal cancer (CRC) transcriptomes and relates to tumor immune-stromal heterogeneity remains incompletely characterized.

**Methods:**

We integrated direct human acrolein target evidence from the Comparative Toxicogenomics Database (CTD) with TCGA-COAD/READ bulk RNA-sequencing profiles and a single-cell RNA-sequencing subset from GSE231559. The CTD curation yielded 179 unique direct human targets. Bulk analyses included 624 primary-tumor and 51 solid-tissue normal profiles. Single-cell analyses included six tumor and three normal libraries, yielding 24,128 retained cells assigned to eight broad marker-supported compartments. Target dysregulation burden, immune-stromal expression programs, and library-aware single-cell target localization were evaluated as transcriptomic summaries. In addition, RT-qPCR was performed in HCT 116 colorectal carcinoma cells to experimentally evaluate four measurable CTD targets (NFKBIA, GSTP1, VIM, and GPX4) after acrolein exposure.

**Results:**

The bulk tumor-normal analysis identified 5,594 differentially expressed genes at FDR < 0.05 and |log2 fold change| ≥ 1. Of 179 CTD acrolein targets, 155 were measurable in the bulk dataset and 43 met the differential-expression threshold. Mean absolute normal-reference target burden was higher in tumors than normal tissue (1.96 vs. 0.75; difference 1.21; Welch *p* = 3.62e-42). Within primary tumors, target burden correlated negatively with T-cell and endothelial programs (rho = −0.27 for each), myeloid (rho = −0.20), fibroblast-ECM (rho = −0.19), and antigen-presentation programs (rho = −0.20), and positively with cell cycle (rho = 0.20). In single-cell data, descriptive mean target scores were highest in myeloid cells (0.455), fibroblasts (0.424), and endothelial cells (0.344). Cross-scale effects for 154 shared targets showed modest concordance (rho = 0.31, *p* = 6.95e-05). RT-qPCR showed vehicle-normalized relative expression of 2.18 ± 0.14 for NFKBIA, 1.83 ± 0.13 for GSTP1, 1.57 ± 0.15 for VIM, and 0.71 ± 0.09 for GPX4 after acrolein exposure (mean ± SD of three biological replicates).

**Conclusions:**

Acrolein target-linked expression perturbation is detectable in CRC and is associated with immune-stromal heterogeneity across bulk and single-cell transcriptomic scales. RT-qPCR provides targeted experimental support for acrolein-responsive expression changes in NFKBIA, GSTP1, VIM, and GPX4 in HCT 116 cells. These findings support a toxicogenomic framework for hypothesis generation but do not measure acrolein exposure in patients, establish causal toxicological effects in human tumors, or demonstrate a specific intercellular signaling mechanism. Additional mechanistic, protein/adduct, dose-response, and independent-cohort validation will be required to determine the biological significance of the observed associations.

## 1 Introduction

Colorectal cancer (CRC) remains a major global malignancy, with approximately 1.93 million new cases and 935,000 deaths estimated worldwide in 2020 (1). Large-scale molecular profiling has established substantial genomic and transcriptomic heterogeneity across colon and rectal cancers (2). Beyond malignant epithelial programs, immune and stromal compartments contribute additional layers of heterogeneity that can influence tumor state and therapeutic response; single-cell studies have further demonstrated lineage-dependent immune and stromal organization in CRC (3).

Acrolein (2-propenal) is a highly reactive alpha,beta-unsaturated aldehyde generated endogenously during lipid peroxidation and encountered exogenously in tobacco smoke, combustion products, and heated foods (4, 5). Its electrophilic reactivity can produce DNA and protein adducts, oxidative stress, mitochondrial injury, and inflammatory perturbation (4). Acrolein has been classified as probably carcinogenic to humans (Group 2A) on the basis of animal carcinogenicity and strong mechanistic evidence (5). In colorectal tissues, acrolein-protein adducts have been reported across stages of colon carcinogenesis (6), and experimental work has linked acrolein exposure to oncogenic transformation and RAS-MAPK activation in CRC-related models and tissues (7).

These observations motivate a transcriptome-level examination of acrolein-related molecular evidence in CRC. The Comparative Toxicogenomics Database (CTD) provides manually curated chemical-gene interactions with traceable literature support (8), enabling a defined target prior that can be integrated with tumor transcriptomes. Cancer-associated fibroblasts and other stromal populations are themselves heterogeneous and can contribute distinct functional states within CRC (9, 10). Public infrastructures such as the NCI Genomic Data Commons and NCBI Gene Expression Omnibus support reproducible integration of bulk and single-cell transcriptomic datasets (11, 12). When combined with immune-stromal expression programs and single-cell localization, this approach can identify where acrolein target-linked dysregulation co-varies with the CRC microenvironment. Importantly, transcriptomic target scores are perturbation proxies rather than measurements of patient acrolein exposure or proof of a causal toxicological pathway.

In this study, we reanalyzed CTD acrolein target evidence together with TCGA-COAD/READ bulk RNA-sequencing data and GSE231559 single-cell RNA-sequencing data. We quantified tumor-normal target dysregulation, examined associations between target burden and eight immune-stromal expression programs, localized measurable targets across broad cell compartments, and assessed descriptive concordance between bulk and single-cell effects. The analysis was designed as an observational toxicogenomic reanalysis with explicit separation between directly curated chemical-gene evidence, observed transcriptomic differences, and exploratory cell-type associations.

## 2 Materials and methods

### 2.1 Database target retrieval

The CTD chemical-gene report was downloaded on the analysis date from https://ctdbase.org/reports/CTD_chem_gene_ixns.tsv.gz and filtered to ChemicalName = Acrolein, chemical identifier D000171, and Organism = Homo sapiens. The report contained 5,550 Acrolein rows overall and 4,799 Homo sapiens rows. After excluding 4,258 co-exposure interaction rows, 541 direct records supported 179 unique primary target genes. Rows were collapsed by gene while preserving CTD actions, number of records, and PubMed identifiers (Python v3.12.5; pandas v2.3.3). Five transparent interpretive modules were used for display: oxidative/electrophile stress, apoptosis/DNA damage, inflammation/immune signaling, barrier/EMT, and cell-cycle control. Module labels are analytic groupings, not additional CTD assertions.

### 2.2 Bulk transcriptome and target-burden proxy

TCGA-COAD/READ count matrices were restricted to primary tumor and solid-tissue normal profiles, retaining 624 tumor patients and 51 normal patients after one profile per patient. Gene-level tumor-normal differential expression used the DESeq2 negative-binomial framework (13) as implemented in PyDESeq2 (14) (PyDESeq2 v0.5.4; Python v3.12.5). Multiple-testing correction used the Benjamini-Hochberg false-discovery-rate procedure (15). For each measured CTD target, expression was standardized against the normal-profile mean and standard deviation. The target dysregulation burden for a sample was the mean absolute normal-reference z score across measured targets; a direction-alignment proxy was separately calculated only for targets with a single CTD expression direction. Both are transcriptomic perturbation summaries (NumPy v2.4.6; pandas v2.3.3; SciPy v1.17.1; scikit-learn v1.9.0; Matplotlib v3.10.9; seaborn v0.13.2).

### 2.3 Immune-stromal heterogeneity

The existing eight marker-based bulk programs (epithelial differentiation, T-cell, cytotoxic, myeloid, fibroblast-ECM, endothelial, antigen presentation, and cell cycle) were joined to the target scores. Spearman correlations were calculated within primary tumors and adjusted across programs by the Benjamini-Hochberg procedure. Tumor target-burden quartiles were shown as descriptive strata (SciPy v1.17.1; pandas v2.3.3; NumPy v2.4.6; Matplotlib v3.10.9; seaborn v0.13.2).

### 2.4 Single-cell processing and localization

The local GSE231559 subset contained six tumor and three normal libraries. Cells with fewer than 300 detected genes or more than 25% mitochondrial counts were excluded, yielding 24,128 retained cells. Counts were library-normalized and log transformed on one combined AnnData object, and downstream single-cell processing and marker scoring were performed with Scanpy (16) (Scanpy v1.12.2; AnnData v0.12.16; Python v3.12.5). Leiden community detection was used for the unsupervised clustering display (17). The shared marker sets were scored with Scanpy score_genes (random_state = 20260825); each cell received the highest-scoring broad label when that score exceeded −0.15. All 24,128 retained cells met that rule and received one of 8 broad labels in the delivered annotation. The resulting cell_id-keyed annotation table was used unchanged for Figures 5–7, so Figure 7 did not perform a second target-specific reannotation. Target and module summaries were calculated at cell level for descriptive displays, then summarized within each library and cell type for contrasts. For cross-scale gene effects, cell-type means were weighted by retained-cell counts to form one mean per gene and library, after which libraries were averaged equally within Tumor and Normal conditions. Thus, condition comparisons use library means and are exploratory because only three normal libraries are available (NumPy v2.4.6; pandas v2.3.3; SciPy v1.17.1; Matplotlib v3.10.9; seaborn v0.13.2).

### 2.5 Experimental RT-qPCR validation

RT-qPCR was performed to experimentally evaluate selected transcriptomic targets in HCT 116 human colorectal carcinoma cells (ATCC CCL-247). Cells were cultured in McCoy's 5A modified medium supplemented with 10% fetal bovine serum at 37 °C and 5% CO2 and were assigned to vehicle control or 10 μM acrolein exposure for 6 h. Four CTD targets represented in the single-cell results—NFKBIA, GSTP1, VIM, and GPX4—were evaluated. The experiment comprised three biological replicates with three technical replicates per biological replicate; technical replicates were averaged before summarizing biological replicates. RT-qPCR reporting was aligned with MIQE principles (18). Relative mRNA expression was calculated by the comparative 2^-ΔΔCt^ method using GAPDH as the reference gene and vehicle as the calibrator (19, 20).

## 3 Results

### 3.1 Bulk cohort quality control

The retained TCGA cohort contained 41 normal and 458 primary-tumor patients in COAD and 10 normal and 166 primary-tumor patients in READ. The panel therefore represents 51 normal and 624 primary-tumor patient profiles across the two projects (Figure 1A). Patient-level eligibility yielded 624 primary patients, 51 normal patients, and 50 patients with both tissue types. Three recurrent or metastatic profiles were excluded from the primary contrast, leaving the displayed sensitivity-set counts (Figure 1B).

**Figure 1:**
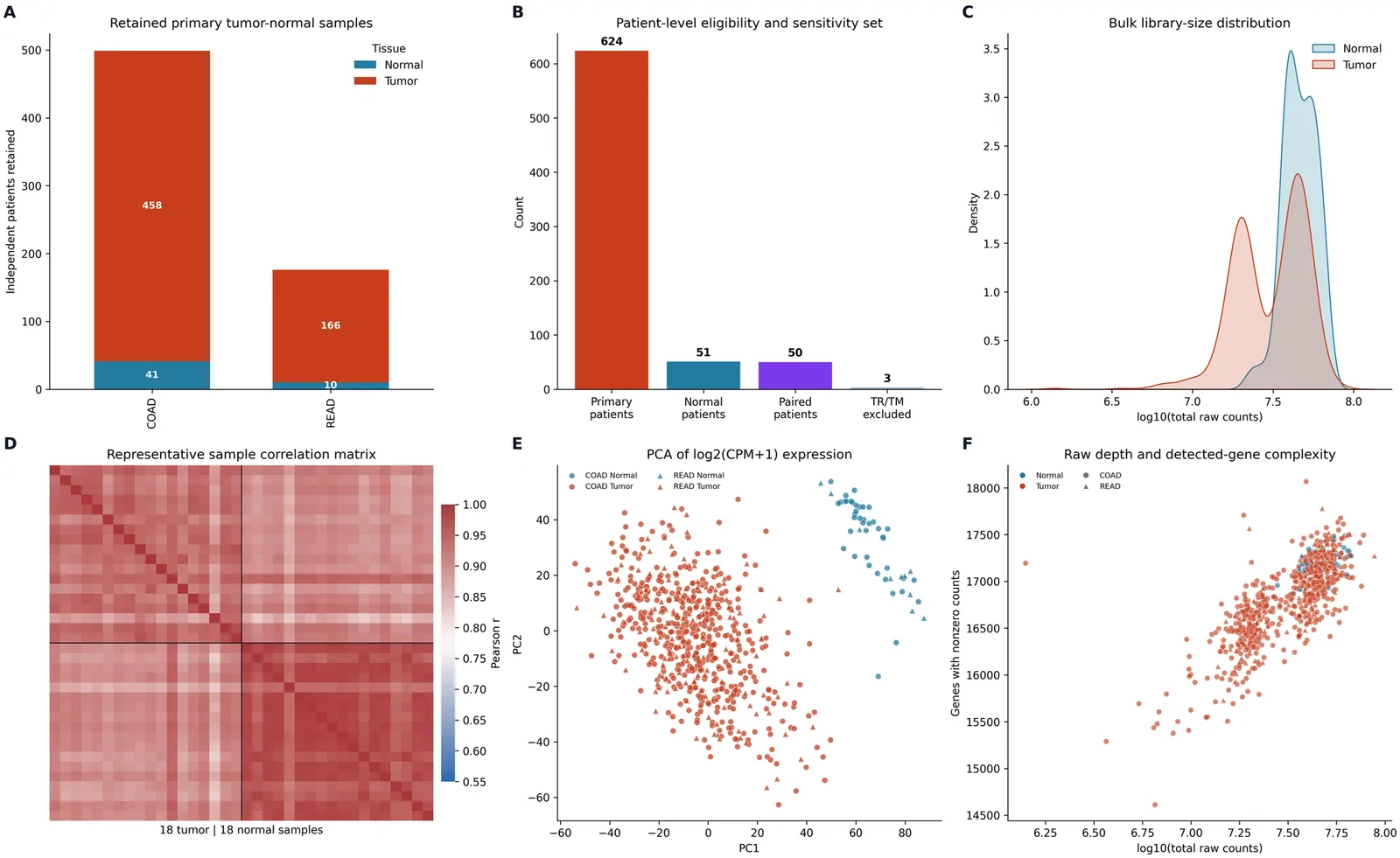
Bulk-transcriptome quality control. **(A)** Retained TCGA profiles by project. **(B)** Patient-level eligibility and sensitivity-set definition. **(C)** Raw bulk library-size distributions. **(D)** Representative sample correlations. **(E)** PCA of log2(CPM + 1) expression. **(F)** Raw sequencing depth versus detected-gene complexity.

Raw library-size distributions were separated by tissue type. Normal libraries were concentrated toward the higher-count mode, whereas tumor libraries showed a broader, partly bimodal distribution; this panel describes input depth rather than biological expression (Figure 1C). The representative correlation matrix showed high within-block sample similarity, with the tumor and normal samples forming visually coherent correlation blocks. The vertical and horizontal dividers mark the 18 tumor and 18 normal samples used for this quality-control display (Figure 1D). PCA of log2(CPM + 1) expression separated normal profiles from the main tumor cloud along the first component, while COAD and READ symbols overlapped within tissue groups. The projection is descriptive and was used to inspect broad sample structure (Figure 1E). Raw total counts and the number of genes with nonzero counts were positively related across libraries. The point shapes retain project identity and the colors retain tissue identity, documenting sequencing-depth and complexity behavior before target scoring (Figure 1F).

### 3.2 Bulk differential expression

The count-aware tumor-normal analysis tested 19,468 genes. Using FDR < 0.05 and absolute log2 fold change ≥ 1, 5,594 genes passed the prespecified threshold, with both up- and down-shifted genes visible in the effect/FDR display (Figure 2A). The MA plot showed that effect estimates were distributed across the abundance range, with greater spread among lower-count genes and a dense central band near zero. Highlighted points identify the thresholded differential-expression subset (Figure 2B). The complete log2 fold-change distribution was centered near zero with asymmetric tails extending in both directions. The shaded interval marks effects between −1 and 1, separating the central background from the prespecified effect threshold (Figure 2C).

**Figure 2:**
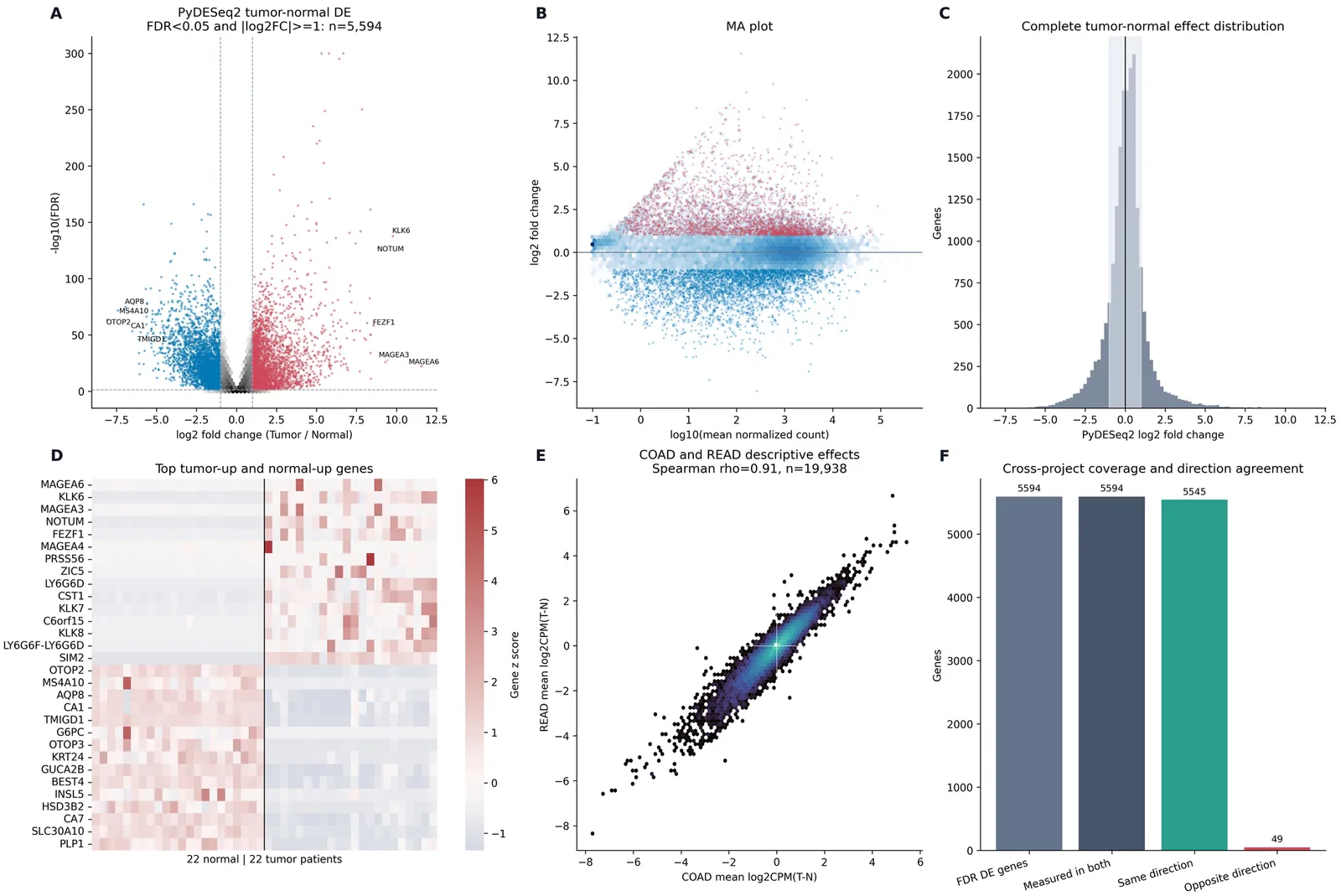
Count-aware differential expression between primary tumors and normal profiles. **(A)** PyDESeq2 effect and FDR display. **(B)** MA plot of effect estimates across mean abundance. **(C)** Complete log2 fold-change distribution. **(D)** Patient-level heatmap of selected genes. **(E)** COAD-READ descriptive effect comparison. **(F)** Cross-project direction agreement.

The patient-level heatmap of the selected tumor-up and normal-up genes showed reciprocal expression patterns across the 22 normal and 22 tumor profiles. The vertical divider separates the two tissue groups and illustrates the sample-level structure behind the gene-level calls (Figure 2D). COAD and READ descriptive gene effects were strongly concordant (Spearman rho = 0.91 across 19,938 genes). The diagonal concentration indicates that the direction and relative magnitude of the broad tumor-normal signal were similar between the two projects (Figure 2E). Among the 5,594 thresholded differential-expression genes, 5,545 had the same direction in COAD and READ and 49 had opposite directions. Thus, most of the selected cross-project signal was directionally consistent, while a small discordant subset remained (Figure 2F).

### 3.3 Bulk immune-stromal programs

The program-score heatmap displayed eight marker-based expression summaries across a representative set of 35 normal and 65 tumor patients. Tumor-normal remodeling was broad, with immune-stromal programs and the cell-cycle program contributing distinct visual patterns (Figure 3A). Tumor-normal distributions differed across programs: cell-cycle scores were shifted upward in tumors, whereas epithelial differentiation, T-cell, cytotoxic, myeloid, fibroblast-ECM, endothelial, and antigen-presentation scores were generally lower in tumors. These are standardized marker scores, not absolute cell fractions (Figure 3B). Bootstrap tumor-minus-normal mean differences were negative for epithelial differentiation (−0.38), T-cell (−0.61), cytotoxic (−0.22), myeloid (−0.86), fibroblast-ECM (−0.20), endothelial (−0.44), and antigen presentation (−0.68), and positive for cell cycle (+1.75); the intervals summarize resampling uncertainty around these descriptive contrasts (Figure 3C).

**Figure 3:**
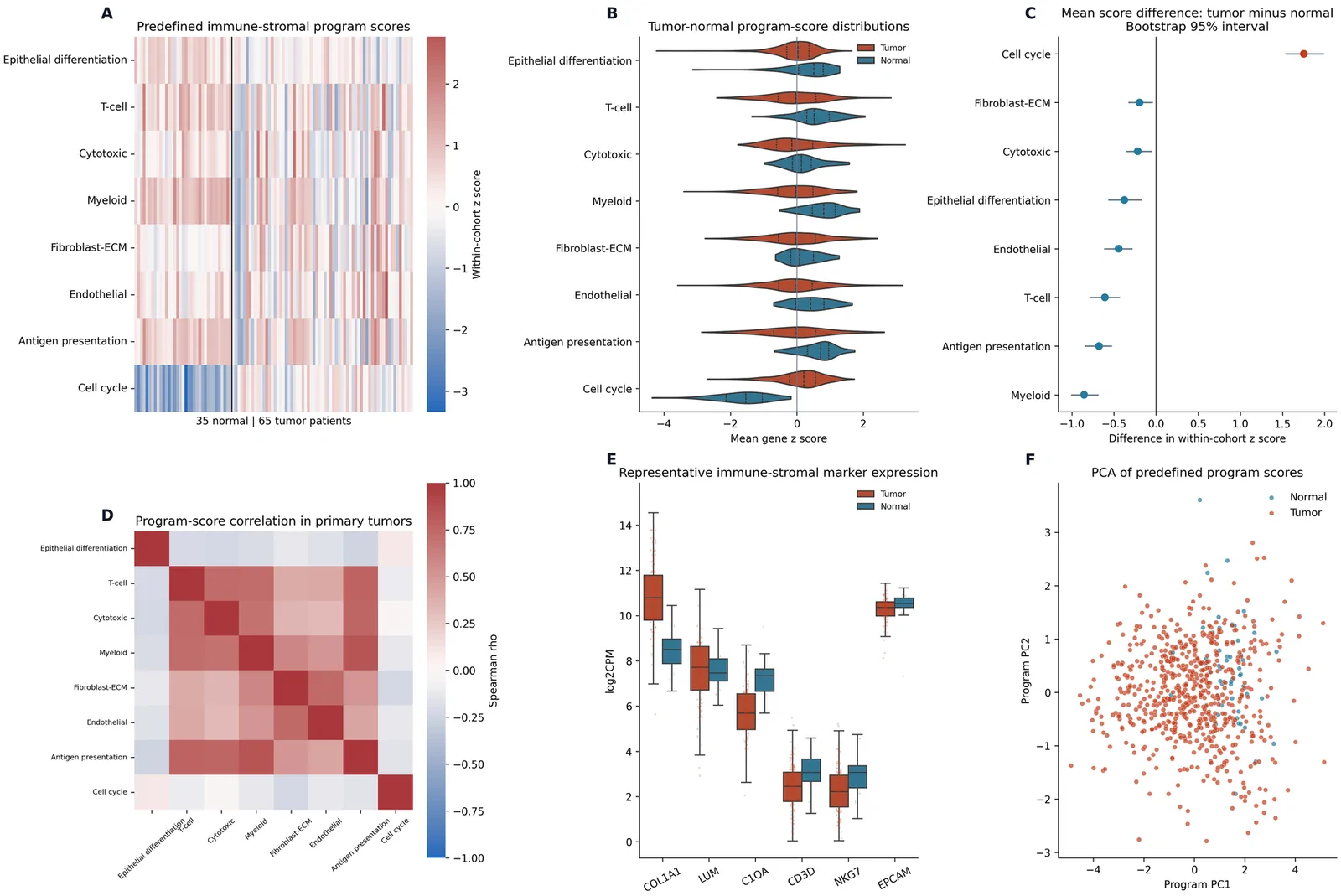
Bulk immune-stromal expression programs. **(A)** Program-score heatmap. **(B)** Tumor-normal distributions. **(C)** Bootstrap program differences. **(D)** Correlations within primary tumors. **(E)** Representative markers. **(F)** Program-score PCA. Scores are marker-based expression summaries, not absolute cell fractions.

Within primary tumors, T-cell, cytotoxic, myeloid, fibroblast-ECM, endothelial, and antigen-presentation programs formed positively correlated groups, while epithelial differentiation and cell cycle were less aligned with the immune-stromal cluster. The matrix describes co-variation among marker summaries (Figure 3D). Representative marker expression retained the expected tissue context: COL1A1 and LUM represented fibroblast-ECM, C1QA represented myeloid cells, CD3D and NKG7 represented lymphoid cytotoxic programs, and EPCAM represented epithelial differentiation. Tumor-normal shifts in these genes provide context for the program scores (Figure 3E). PCA of the eight program scores showed a broad tumor cloud and a smaller normal set with partial overlap. The projection summarizes multivariate program remodeling and is not a classifier of individual samples (Figure 3F).

### 3.4 Acrolein target evidence and bulk perturbation

The CTD report contained 5,550 Acrolein records; the primary set comprised 179 unique direct human target genes. The display grouped them into 92 other CTD targets, 24 oxidative/electrophile-stress targets, 23 inflammation/immune-signaling targets, 23 apoptosis/DNA-damage targets, 13 barrier/EMT targets, and 4 cell-cycle-control targets (Figure 4A). CTD direction evidence was heterogeneous: 73 target genes had increased expression evidence, 30 had decreased expression evidence, 5 had mixed directions, and 71 were supported by non-expression actions. The categories were retained as recorded evidence and were not forced into a single signed expression score (Figure 4B). Of the 179 CTD targets, 155 were measured in the bulk transcriptome and 43 met the base differential-expression threshold (23 higher and 20 lower in tumors). Their locations in the full effect/FDR background show that the curated prior was not uniformly shifted (Figure 4C).

**Figure 4:**
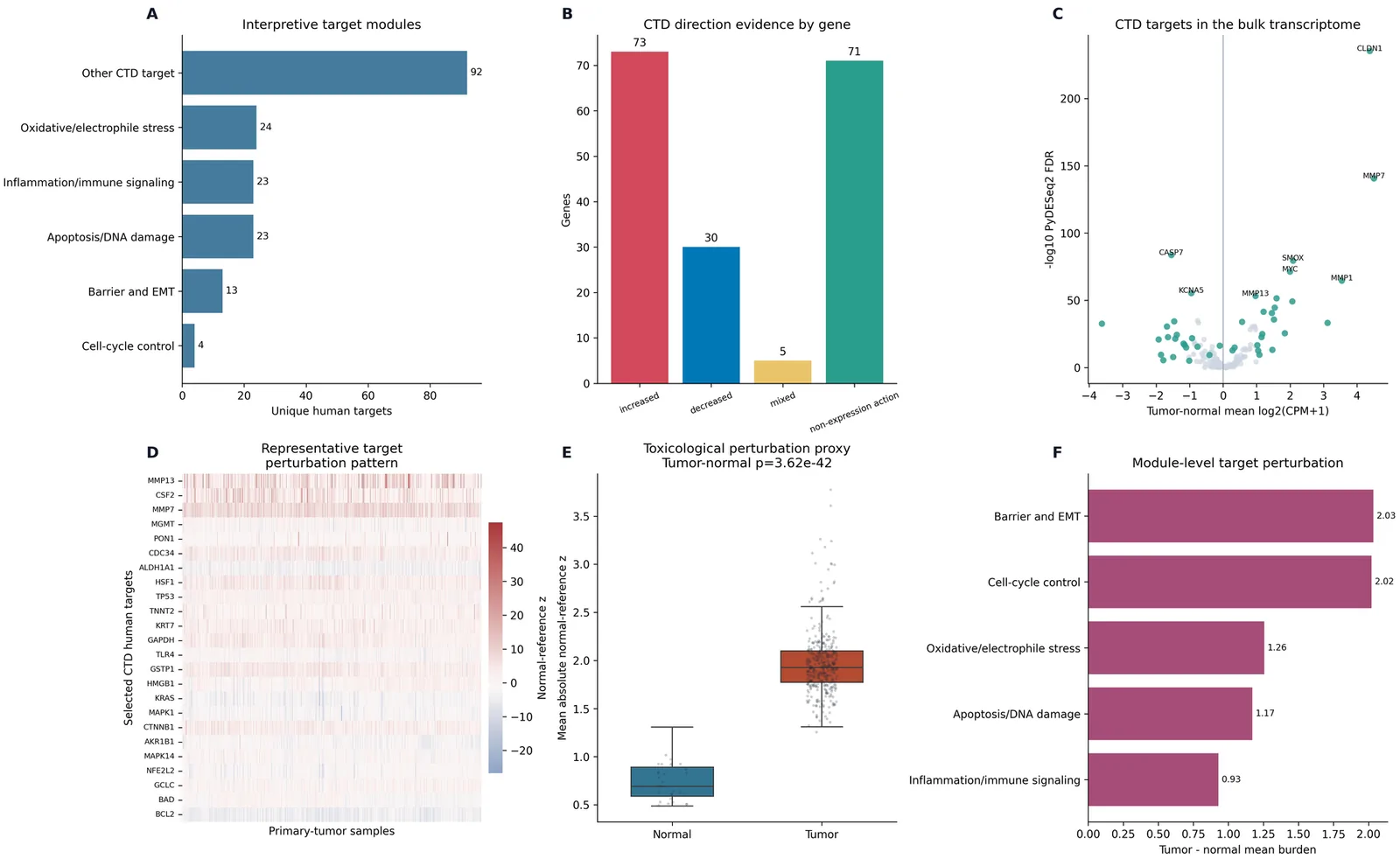
Acrolein target evidence and bulk toxicological perturbation. **(A)** Interpretive target modules. **(B)** CTD direction evidence by gene. **(C)** Measured CTD targets in the bulk differential-expression background. **(D)** Representative high-variance target perturbation pattern in primary tumors. **(E)** Tumor-normal comparison of the toxicological perturbation proxy. **(F)** Module-level tumor-normal target perturbation differences.

The normal-reference heatmap of selected CTD targets showed heterogeneous perturbation across primary tumors. Individual targets displayed different amplitudes and directions, so the panel supports a distributed perturbation pattern rather than a uniform target-set response (Figure 4D). The mean absolute normal-reference target burden was higher in primary tumors than normals (1.96 versus 0.75; difference 1.21; Welch *p* = 3.62e-42). This score is an expression-based toxicological perturbation proxy relative to normal tissue (Figure 4E). All five prespecified modules had positive tumor-minus-normal burden differences: oxidative/electrophile stress 1.26, apoptosis/DNA damage 1.17, inflammation/immune signaling 0.93, barrier/EMT 2.03, and cell-cycle control 2.02. The largest differences were observed for barrier/EMT and cell-cycle control (Figure 4F).

### 3.5 Single-cell quality control and atlas

The local GSE231559 subset contained nine libraries. Barcode input and post-QC retention varied substantially by library, and the retained library counts summed to 24,128 cells; the panel preserves library-level rather than pooled-cell accounting (Figure 5A). Post-QC cells followed the expected increasing relationship between total counts and detected genes. Mitochondrial percentage varied across the cloud, providing a visual check of the retained-cell complexity after the fewer-than-300-genes and greater-than-25%-mitochondrial-count filters (Figure 5B). The Leiden UMAP resolved multiple transcriptional clusters across the retained cells. Cluster colors are an unsupervised embedding display and do not by themselves define biological cell types (Figure 5C).

**Figure 5:**
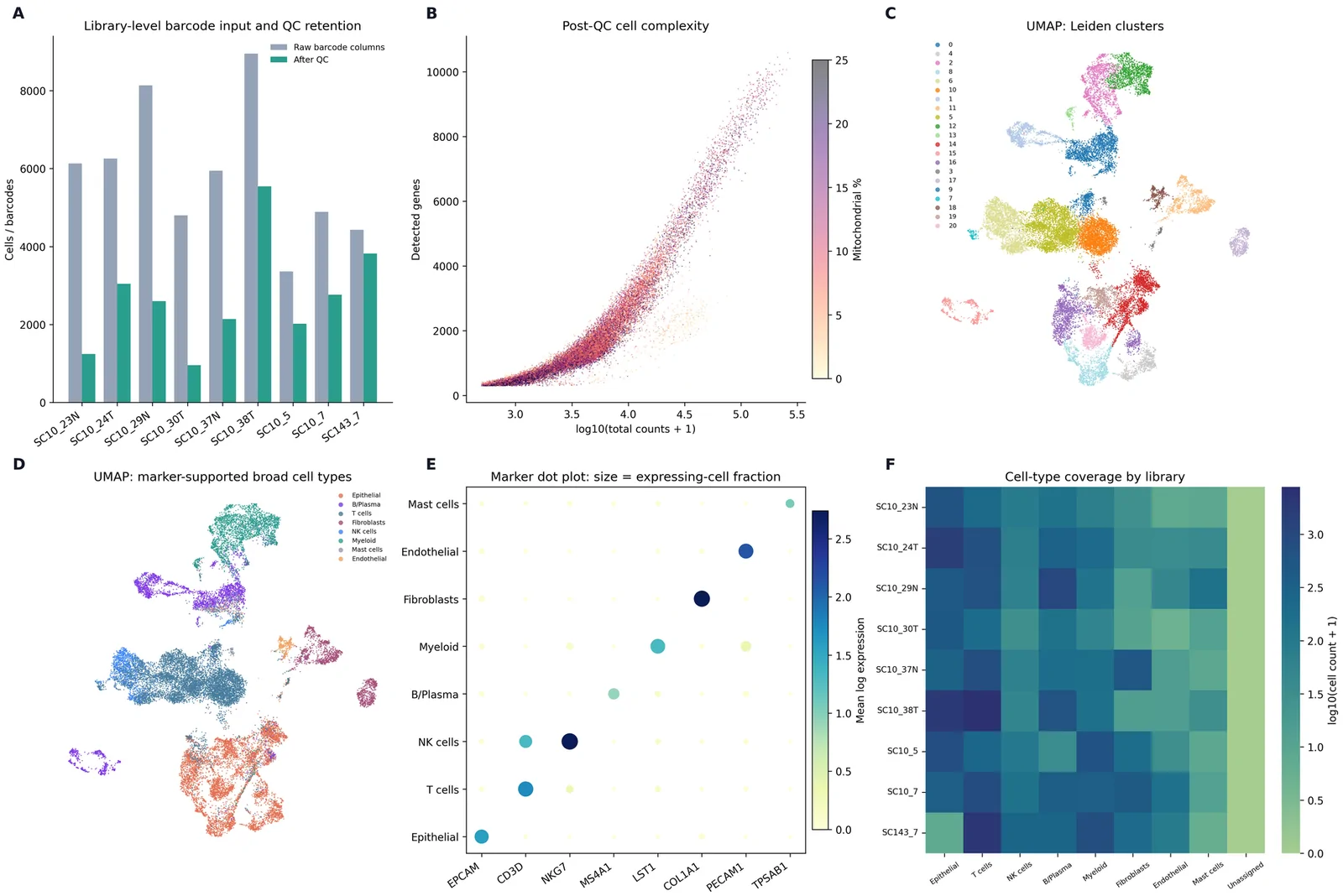
Single-cell quality control and marker-supported atlas. **(A)** Library barcode input and QC retention. **(B)** Post-QC cell complexity. **(C)** Leiden UMAP. **(D)** Broad marker-supported cell types. **(E)** Marker dot plot. **(F)** Cell-type coverage by library. Cell-level displays are descriptive.

Marker-supported broad labels mapped the UMAP into the following compartments: Epithelial, T cells, NK cells, B/Plasma, Myeloid, Fibroblasts, Endothelial, and Mast cells. These labels provide the cellular frame used for subsequent target localization (Figure 5D). The marker dot plot showed concordant marker localization: EPCAM in epithelial cells, CD3D in T cells, NKG7 in NK cells, MS4A1 in B/plasma cells, LST1 in myeloid cells, COL1A1 in fibroblasts, PECAM1 in endothelial cells, and TPSAB1 in mast cells (Figure 5E). Cell-type coverage was uneven across libraries. The heatmap therefore documents which broad compartments were represented in each library and indicates where library-level contrasts may be less stable because of sparse coverage (Figure 5F).

### 3.6 Library-aware tumor-immune context

Within-library composition differed markedly among the nine libraries. Epithelial, T-cell, B/plasma, myeloid, and fibroblast shares varied by library, demonstrating why pooled-cell proportions were not treated as a single cohort-level composition estimate (Figure 6A). The three matched normal-to-tumor pairs (C1-C3) showed pair-specific changes in epithelial, myeloid, fibroblast, T-cell, and NK-cell shares. Each line connects two libraries from one patient pair, so the panel is descriptive of matched composition changes (Figure 6B). Library-mean tumor-minus-normal composition differences were positive for myeloid (+0.072), T cells (+0.053), epithelial cells (+0.022), and endothelial cells (+0.009), and negative for NK cells (−0.002), mast cells (−0.015), fibroblasts (−0.039), and B/plasma cells (−0.098). These differences are descriptive and depend on the available library coverage (Figure 6C).

**Figure 6:**
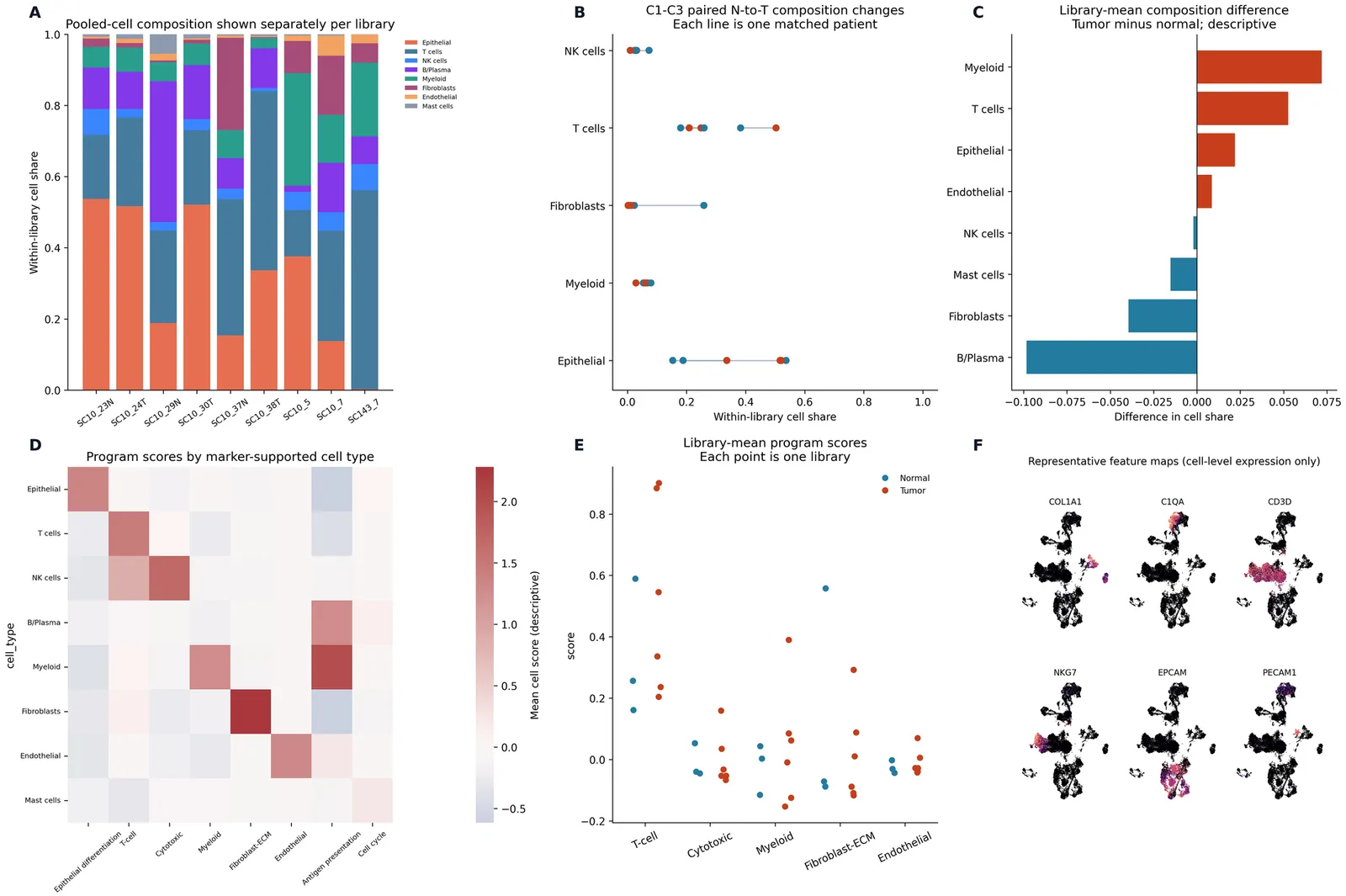
Library-aware tumor-immune microenvironment context. **(A)** Within-library composition. **(B)** C1-C3 matched-pair composition. **(C)** Library-mean composition differences. **(D)** Cell-type program localization. **(E)** Library-mean program scores. **(F)** Representative feature maps. These panels do not infer causal signaling.

Program scores localized to marker-supported compartments: fibroblast-ECM scores were highest in fibroblasts, myeloid scores in myeloid cells, cytotoxic scores in NK cells, and T-cell scores in T cells. The heatmap is a cell-type context display rather than a causal signaling map (Figure 6D). Library-mean program scores varied across the six tumor and three normal libraries. Each point is one library, making the panel explicit about the inferential unit and about the limited number of normal libraries (Figure 6E). Feature maps for COL1A1, C1QA, CD3D, NKG7, EPCAM, and PECAM1 placed representative marker expression on the shared cellular embedding. The maps provide spatial expression context for the broad labels without implying intercellular signaling (Figure 6F).

### 3.7 Single-cell acrolein target localization

Cell-level target scores spanned the eight marker-supported compartments, with descriptive means ordered as Myeloid (0.455), Fibroblasts (0.424), Endothelial (0.344), Epithelial (0.330), NK cells (0.269), T cells (0.244), Mast cells (0.232), B/Plasma (0.230). Cells are display units here, not independent biological replicates (Figure 7A). The 154 measurable CTD targets were detectable across the following compartments: Epithelial, T cells, NK cells, B/Plasma, Myeloid, Fibroblasts, Endothelial, and Mast cells. The heatmap of the top expressed measured targets showed compartment-specific intensity patterns rather than confinement to one lineage (Figure 7B). Library-level target scores varied across the nine libraries: normal libraries ranged from 0.219 to 0.308, while tumor libraries ranged from 0.249 to 0.428. Because each point is a library and the normal group contains only three libraries, these values support localization and variation rather than a definitive condition effect (Figure 7C).

**Figure 7:**
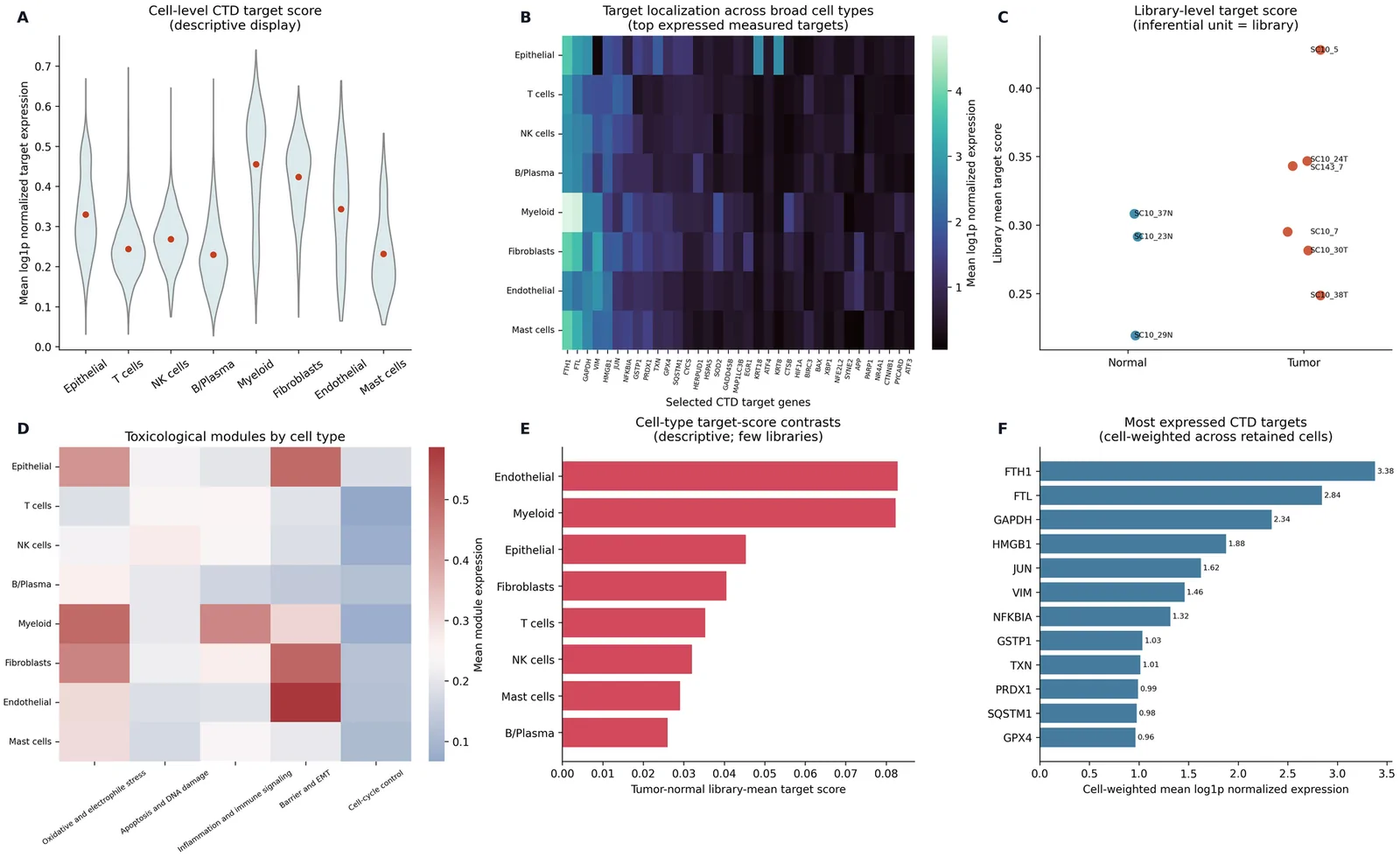
Acrolein target localization in the colorectal tumor-immune microenvironment. **(A)** Cell-level target-score distributions, shown descriptively. **(B)** Target expression for the top expressed measured targets across broad cell types. **(C)** Library-level target scores, with library as the inferential unit. **(D)** Interpretive toxicological modules by cell type. **(E)** Exploratory library-level cell-type contrasts. **(F)** Cell-count-weighted mean expression of the most expressed measured CTD targets across retained cells. Cell-type labels in Figures 5−7 are inherited from the same combined-AnnData Scanpy marker-scoring workflow; Figure 7 uses no target-specific reannotation.

Module scores differed by cell type. Barrier/EMT values were particularly high in endothelial, epithelial, and fibroblast compartments, inflammation/immune-signaling values were relatively high in myeloid and mast cells, and apoptosis/DNA-damage and oxidative modules were detectable across several compartments (Figure 7D). Exploratory library-mean target-score contrasts were positive for all displayed broad cell types, with the largest differences for Endothelial (+0.083) and Myeloid (+0.082); the corrected contrasts were not compelling with the limited library counts. The panel is therefore descriptive/exploratory (Figure 7E). After retained-cell weighting, the most expressed CTD targets were FTH1 (3.38), FTL (2.84), GAPDH (2.34), HMGB1 (1.88), JUN (1.62), VIM (1.46), NFKBIA (1.32), GSTP1 (1.03), TXN (1.01), PRDX1 (0.99), SQSTM1 (0.98), GPX4 (0.96). These values summarize expression across all 24,128 retained cells (Figure 7F).

### 3.8 Experimental RT-qPCR validation

In HCT 116 cells, vehicle-normalized relative expression after acrolein exposure was NFKBIA 2.18 ± 0.14-fold, GSTP1 1.83 ± 0.13-fold, VIM 1.57 ± 0.15-fold, and GPX4 0.71 ± 0.09-fold (mean ± SD of three biological replicates; Figure 8). Thus, NFKBIA, GSTP1, and VIM showed increased relative mRNA expression, whereas GPX4 showed decreased relative expression under the tested condition. These experimental RT-qPCR data provide targeted validation of the selected transcriptional responses but, because only four genes and one exposure condition were tested, they do not establish a genome-wide or patient-level acrolein effect.

**Figure 8:**
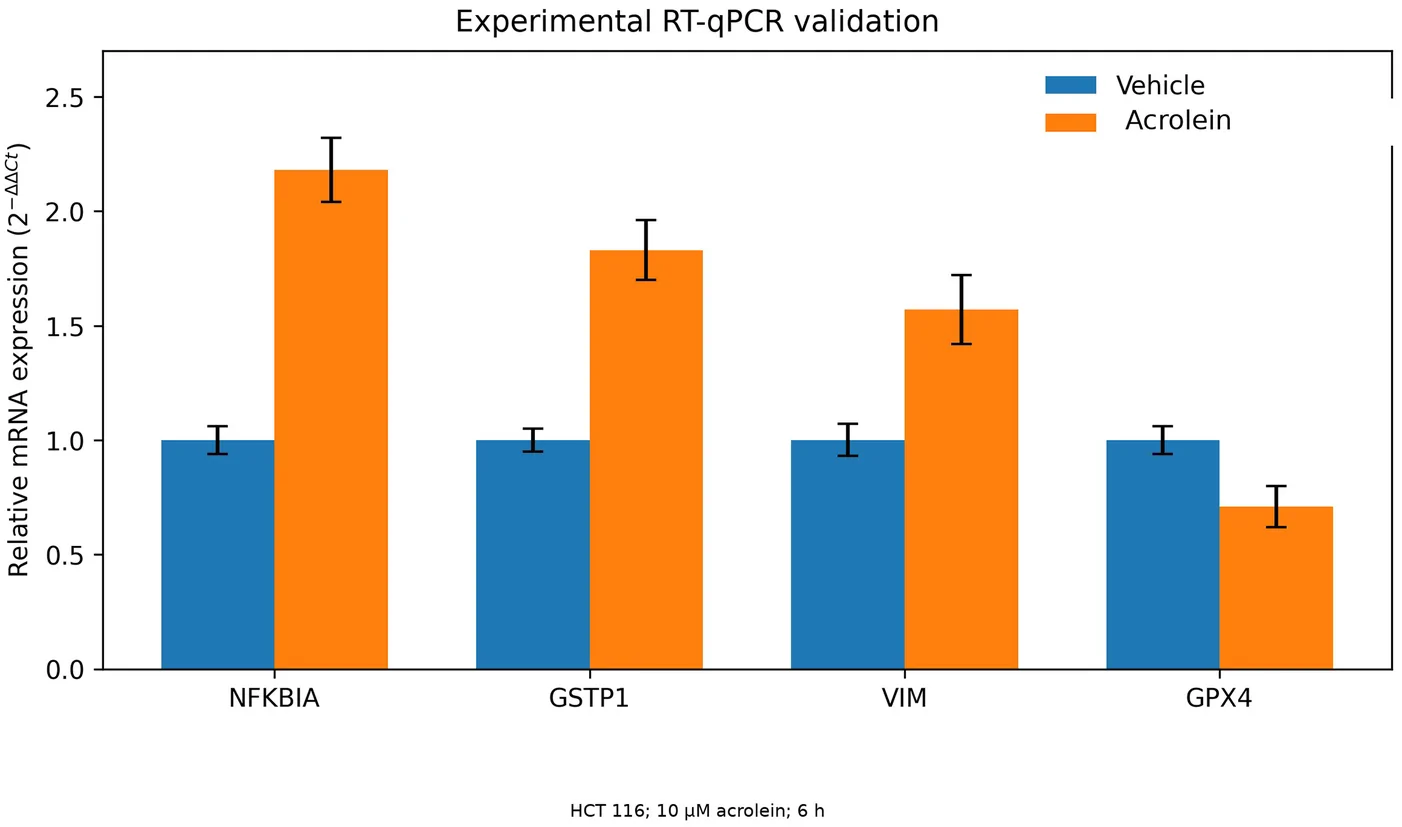
Experimental RT-qPCR validation of four selected acrolein-related targets in HCT 116 colorectal carcinoma cells (ATCC CCL-247). Bars show vehicle-normalized relative mRNA expression for vehicle control and 10 μM acrolein exposure for 6 h for NFKBIA, GSTP1, VIM, and GPX4. Values are mean ± SD for three biological replicates, with technical triplicates averaged within each biological replicate. Relative expression was calculated using GAPDH as the reference gene and the comparative 2^-ΔΔCt^ method.

### 3.9 Integrated target burden and heterogeneity

Across primary tumors, Acrolein target burden correlated negatively with the fibroblast-ECM program (Spearman rho = −0.19, *p* = 2.97e-06). This is an association between transcriptomic summaries and does not establish fibroblast depletion or a causal toxicological pathway (Figure 9A). Target burden correlations with the eight bulk programs were −0.16 for epithelial differentiation, −0.27 for T-cell, −0.12 for cytotoxic, −0.20 for myeloid, −0.19 for fibroblast-ECM, −0.27 for endothelial, −0.20 for antigen presentation, and +0.20 for cell cycle. The panel summarizes heterogeneity axes within primary tumors (Figure 9B). Tumors grouped by low, middle, and high target burden showed graded differences in T-cell, cytotoxic, myeloid, fibroblast-ECM, and antigen-presentation scores. Lower-burden tumors tended to have more positive immune-stromal scores than high-burden tumors in this display; the groups are descriptive quartile strata rather than clinical categories (Figure 9C).

**Figure 9:**
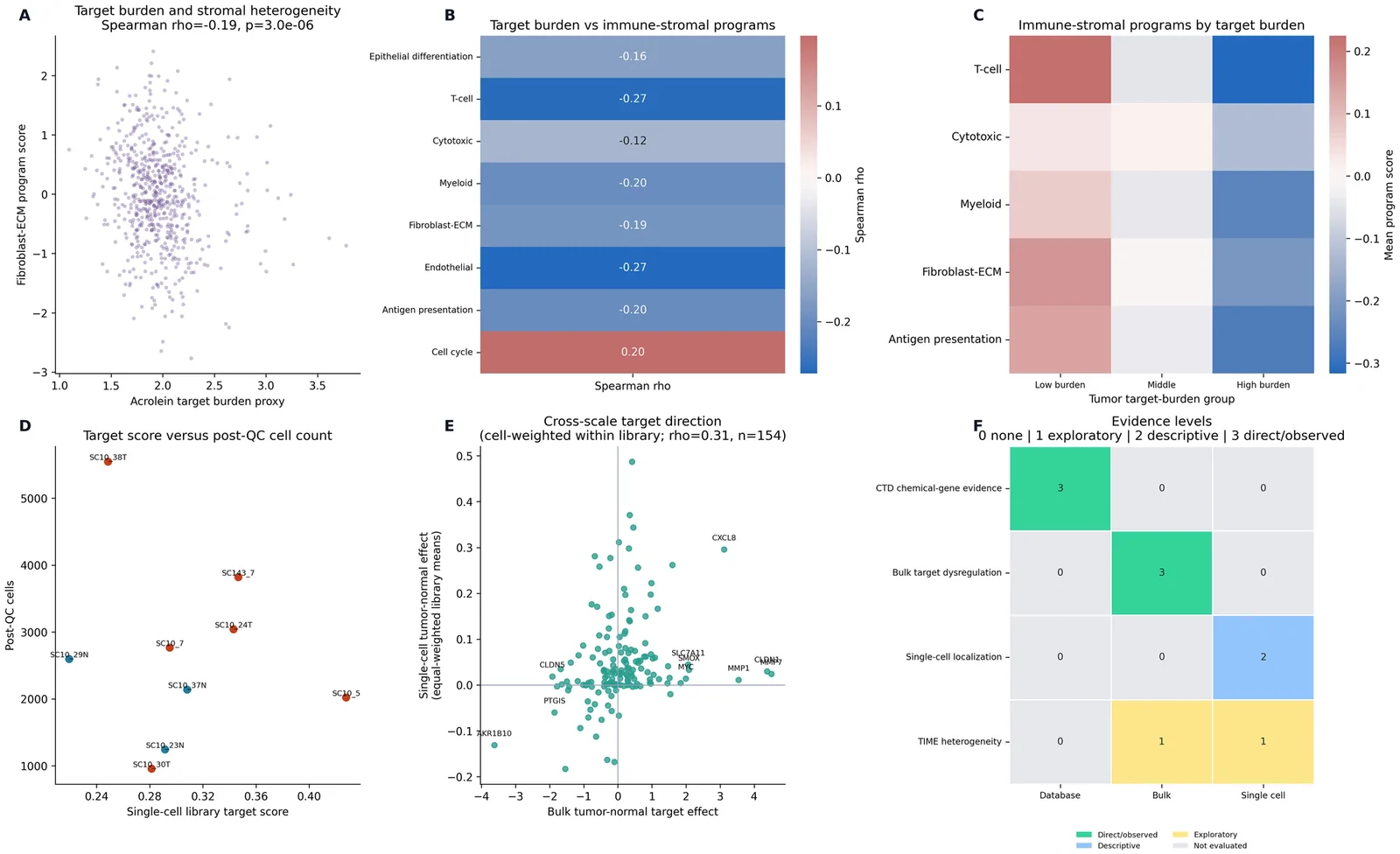
Integrated acrolein perturbation proxy and tumor-immune heterogeneity. **(A)** Target burden versus fibroblast-ECM heterogeneity. **(B)** Target burden correlations with immune-stromal programs. **(C)** Program scores across tumor target-burden strata. **(D)** Single-cell library target score versus post-QC cell count (sampling depth). **(E)** Cross-scale target direction using retained-cell-count-weighted within-library gene means and equal library weighting within condition. **(F)** Evidence levels: green = direct/observed, blue = descriptive, amber = exploratory, and gray = not evaluated; direct/observed does not imply causality.

Single-cell library target score was plotted against post-QC cell count for all nine libraries. The spread of points shows that target score and retained-cell yield varied independently enough that cell count should be treated as a sampling-depth descriptor, not as cell-type composition (Figure 9D). Bulk tumor-normal target effects and single-cell effects based on retained-cell-count-weighted means within each library and equal weighting of libraries within condition were available for 154 shared targets. Their rank correlation was rho = 0.31 (*p* = 6.95e-05), providing cross-scale descriptive concordance (Figure 9E). The evidence matrix classified CTD chemical-gene evidence and bulk target dysregulation as direct/observed, single-cell localization as descriptive, and TIME heterogeneity as descriptive or exploratory depending on the data layer. The labels define claim boundaries; direct/observed does not mean causal (Figure 9F).

## 4 Discussion

This integrative reanalysis identifies a reproducible pattern of acrolein target-linked transcriptomic perturbation in CRC while maintaining clear boundaries on causal interpretation. Among 179 direct human CTD targets, 155 were measurable in the TCGA bulk dataset and 43 met the prespecified differential-expression threshold. The mean absolute normal-reference target burden was substantially higher in primary tumors than normal tissue (1.96 vs. 0.75), and all five prespecified interpretive target modules showed positive tumor-minus-normal burden differences. These results indicate distributed dysregulation of curated acrolein-responsive genes rather than a uniform directional response.

The transcriptomic findings should be interpreted as target-linked toxicogenomic context rather than evidence of acrolein exposure in the analyzed patients. Acrolein is a reactive electrophile capable of DNA and protein adduction, oxidative stress, and broader cellular injury (4), and its carcinogenic hazard has been recognized by IARC (5). Evidence from human colon tissue and experimental CRC models also supports a relationship between acrolein-related damage and colorectal tumorigenesis (6, 7). However, the target-burden metric is derived solely from transcript abundance relative to normal-reference expression. It therefore cannot determine the source, dose, or timing of acrolein exposure in the TCGA or GSE231559 samples and does not establish that acrolein caused the observed tumor transcriptional state.

Within primary tumors, higher target burden was associated with lower T-cell, cytotoxic, myeloid, fibroblast-ECM, endothelial, and antigen-presentation program scores and with a higher cell-cycle score. These are correlations among standardized expression summaries rather than estimates of absolute cell fractions. The pattern is consistent with the broader observation that CRC immune and stromal organization varies substantially across tumors (3). In particular, fibroblast and immune programs are biologically heterogeneous and can reflect multiple cell states and functions rather than a single pro- or antitumor phenotype (9, 10). Accordingly, the negative bulk correlations should be viewed as axes of co-variation that generate hypotheses for follow-up rather than evidence of immune exclusion, stromal depletion, or a specific signaling pathway.

The single-cell analysis provides complementary localization rather than mechanistic validation. Descriptive mean target scores were highest in myeloid cells (0.455), fibroblasts (0.424), and endothelial cells (0.344), while the 154 measurable CTD targets were distributed across all eight broad compartments. Exploratory library-mean tumor-normal target-score contrasts were positive across the displayed cell types, but corrected contrasts were not compelling given the small number of libraries, particularly the three normal libraries. The apparently high cell-level target score in fibroblasts and the negative bulk correlation between target burden and the fibroblast-ECM program (rho = −0.19) are not contradictory: the former describes target localization across cell compartments, whereas the latter measures between-tumor co-variation between two sample-level summaries.

Cross-scale comparison of 154 shared targets yielded modest but statistically detectable rank concordance between bulk and single-cell effects (rho = 0.31, *p* = 6.95e-05). This degree of agreement supports partial consistency across data scales while also emphasizing model and sampling differences. The bulk analysis represents hundreds of patient-level profiles, whereas the single-cell component contains nine libraries with unequal cell-type coverage. Therefore, the single-cell results are most appropriately used to contextualize localization and heterogeneity, not to claim a unique cellular origin or a causal cell-cell communication mechanism.

The RT-qPCR experiment adds an orthogonal *in vitro* validation layer to the computational analyses. Under the tested 10 μM acrolein, 6 h condition, NFKBIA, GSTP1, and VIM increased relative to vehicle, whereas GPX4 decreased. This mixed directional response is compatible with the study's broader conclusion that acrolein-linked targets do not behave as a uniformly shifted gene set. The qPCR component was reported according to established quantitative-PCR guidance (18) and analyzed with the comparative Ct framework (19, 20). Its role is targeted experimental support for four prespecified genes, not confirmation that the transcriptomic target-burden proxy directly measures acrolein exposure in human CRC.

Several limitations should be considered. First, the bulk and single-cell components are secondary analyses of public transcriptomic datasets and curated CTD records, without direct measurement of acrolein or acrolein-derived adducts in those patient samples. Second, the normal-reference target burden is an expression-based proxy whose magnitude can be influenced by tumor composition and transcriptional state. Third, the single-cell dataset contains only three normal libraries, and broad marker-based annotation may not resolve finer functional subtypes. Fourth, the bulk and single-cell datasets differ in platform, sampling structure, and inferential unit, as reflected by the modest cross-scale correlation. Fifth, although the RT-qPCR component provides direct experimental perturbation data, it is limited to four targets in one colorectal cancer cell line at a single acrolein concentration and time point. Additional dose- and time-response experiments, independent cell models, protein and acrolein-adduct measurements, functional assays, spatial validation, and independent CRC cohorts will be needed to define mechanism and translational relevance.

## 5 Conclusions

Integrating CTD chemical-gene evidence with TCGA bulk transcriptomes and GSE231559 single-cell data shows that acrolein target-linked genes are heterogeneously perturbed in CRC and that the resulting target-burden proxy co-varies with immune-stromal expression programs. Single-cell analysis localizes measurable targets across multiple compartments, with relatively high descriptive scores in myeloid cells, fibroblasts, and endothelial cells, while cross-scale concordance remains modest. Experimental RT-qPCR in HCT 116 cells showed increased NFKBIA, GSTP1, and VIM expression and decreased GPX4 expression after acrolein exposure, providing targeted experimental support for the selected genes. Together, these findings define a hypothesis-generating toxicogenomic framework rather than a clinical risk model or a demonstrated causal mechanism in human tumors. Further mechanistic and independent-cohort validation remains necessary.

## Data Availability

The publicly available datasets analyzed in this study are available from the Genomic Data Commons (TCGA-COAD and TCGA-READ), the Gene Expression Omnibus (GSE231559), and the Comparative Toxicogenomics Database (CTD). The raw RT-qPCR data generated in this study are available from the corresponding author on reasonable request.
